# Teaching clinical reasoning to undergraduate medical students by illness script method: a randomized controlled trial

**DOI:** 10.1186/s12909-021-02522-0

**Published:** 2021-02-02

**Authors:** Mana Moghadami, Mitra Amini, Mohsen Moghadami, Bhavin Dalal, Bernard Charlin

**Affiliations:** 1 Shiraz Medical School, Shiraz University of Medical Sciences, Shiraz, Iran; 2 Clinical Education Research Center, Shiraz University of Medical Sciences, Shiraz, Iran; 3 Foundation for Advancement of International Medical Education and Research (FAIMER), Philadelphia, USA; 4 Noncommunicable Disease Research Center, Shiraz University of Medical Sciences, Shiraz, Iran; 5 Oakland University William Beaumont School of Medicine, Rochester, MI USA; 6 Medical School, University of Montreal, Montreal, Canada

**Keywords:** Students, Medical, Education, Clinical reasoning, Script concordance test, Illness script

## Abstract

**Background:**

The illness script method employs a theoretical outline (e.g., epidemiology, pathophysiology, signs and symptoms, diagnostic tests, interventions) to clarify how clinicians organized medical knowledge for clinical reasoning in the diagnosis domain. We hypothesized that an educational intervention based on the illness script method would improve medical students’ clinical reasoning skills in the diagnosis domain.

**Methods:**

This study is a randomized controlled trial involving 100 fourth-year medical students in Shiraz Medical School, Iran. Fifty students were randomized to the intervention group, who were taught clinical reasoning skills based on the illness script method for three diseases during one clinical scenario. Another 50 students were randomized to the control group, who were taught the clinical presentation based on signs and symptoms of the same three diseases as the intervention group. The outcomes of interest were learner satisfaction with the intervention and posttest scores on both an internally developed knowledge test and a Script Concordance Test (SCT).

**Results:**

Of the hundred participating fourth-year medical students, 47 (47%) were male, and 53 (53%) were female. On the knowledge test, there was no difference in pretest scores between the intervention and control group, which suggested a similar baseline knowledge in both groups; however, posttest scores in the intervention group were (15.74 ± 2.47 out of 20) statistically significantly higher than the control group (14.38 ± 2.59 out of 20, *P* = 0.009). On the SCT, the mean score for the intervention group (6.12 ± 1.95 out of 10) was significantly higher than the control group (4.54 ± 1.56 out of 10; *P* = 0.0001). Learner satisfaction data indicated that the intervention was well-received by students.

**Conclusion:**

Teaching with the illness script method was an effective way to improve students’ clinical reasoning skills in the diagnosis domain suggested by posttest and SCT scores for specific clinical scenarios. Whether this approach translates to improved generalized clinical reasoning skills in real clinical settings merits further study.

**Supplementary Information:**

The online version contains supplementary material available at 10.1186/s12909-021-02522-0.

## Background

Physicians use clinical reasoning skills to gather patient data, combine it with their previous knowledge, and then make a clinical impression, diagnosis, and management plan [1, 2]. It is a critical skill in caring for patients effectively and efficiently and must be a part of every health professional education curriculum [3]. However, according to a survey of 123 United States internal medicine clerkship directors, medical students lack clinical reasoning concepts. The authors recommended that all undergraduate medical education curriculum incorporate a structured curriculum in the clinical reasoning field [4].

The conceptual framework of script theory holds that human brains interpret the world by comparing the features of the mental models it makes with a real scene’s structures, checking for consistencies and inconsistencies, patterns, and irregularities. Based on the script theory, expert clinicians make a diagnosis by considering related differential diagnoses based on comparing and contrasting key features. These expert clinicians activate networks of prearranged knowledge, called “illness scripts” [5]. The illness script method uses a theoretical outline to clarify how medical diagnostic knowledge will be organized into different categories. These categories include epidemiology, the pathophysiology of diseases, symptoms, clinical signs, and interventions, leading to an accurate diagnosis [6].

As novice learners, most medical students currently develop clinical reasoning skills informally in clinical wards with varying degrees of supervision. They generally organize their medical knowledge according to the components of the curriculum. When making a diagnosis, medical students often use a process of hypothesis generation and try to test one symptom at a time.

Teaching clinical reasoning by illness script model could help medical students acquire acceptable skills in generating differential diagnoses and clinical data interpretations [7].

Different assessment methods like clinical scenario-based multiple-choice questions, extended matching questions, and well-known clinical reasoning tests, such as the Script Concordance Test (SCT), may provide reliable evidence of medical students’ clinical reasoning skills in the diagnosis domain [8]. SCT is one assessment of clinical reasoning skills that emphasizes data interpretation by asking learners to estimate the impact of new information on a suggested hypothesis. The SCT is based on the illness script method, which was developed in the field of cognitive psychology. The SCT measures the progress of illness scripts method in medical students as novice learners by comparing their performance on this test to a panel of expert physicians [9].

To better prepare the students for clinical rotations, we designed a brief educational intervention around clinical reasoning in the diagnosis domain. We hypothesized that an education intervention based on the illness script method would improve students’ clinical reasoning skills in the diagnosis domain.

## Methods

### Study design and participants

This Ranomized controlled trial was conducted at Shiraz Medical School, which was established in 1952 in south Iran. The medical school has a seven-year undergraduate medical education curriculum, including horizontal integration of basic science courses and 36 months of clinical rotations. Graduates are qualified for medical practice as general practitioners, but they may continue their education in specialties and subspecialties [10, 11].

The sample size had been calculated to be 32 in each group, assuming a power of 90%, the confidence interval of 95%, standard deviation 5.89, and 4.04 to find a 3.6 difference in the groups’ mean. Regarding at least 20% of the missing, this number had increased to 50 in each group.

**Fig. 1:**
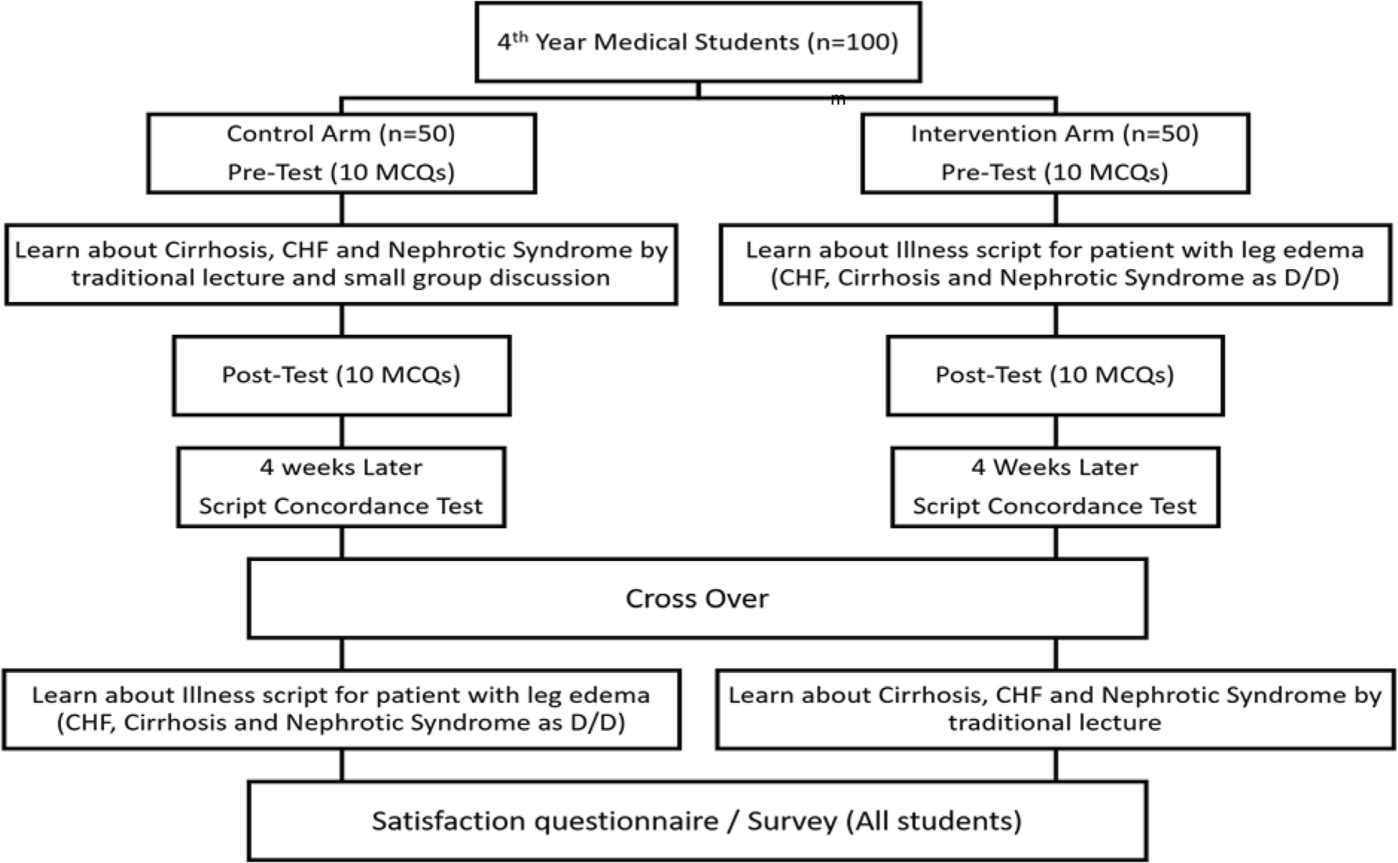
Diagram of the study design

A total of hundred fourth-year medical students were selected randomly from all students who entered the didactic classes in the internal medicine department to participate in the randomized controlled trial. Fifty students were randomized to either the control group or the intervention group. We used the CONSORT statement about randomized controlled trials [12]. A diagram of the study design is shown in Fig. 1. For ethical purposes, after the initial intervention and measurements of outcomes, students crossed over to the other group to ultimately receive the educational content in both formats. Both groups attended teaching sessions (workshops) that lasted around 7 h, excluding breaks. Both workshops were guided by an internal medicine expert who was highly familiar with teaching clinical reasoning skills in the diagnosis domain (third author). Several tutors also helped during small group sessions during both workshops. Descriptions, timings, and agendas of each group’s workshops are described in Table 1.

**Table 1:** The description of intervention and control group’s workshop

Component	Time (Minutes)	Intervention Group	Control Group
**Introduction**	30	Didactic lecture Essential components of the clinical diagnostic process	Didactic lecture Edema
**Main Topic**	180	Problem representation and developing illness script	Lecturing three diseases (Cirrhosis, CHF, and Nephrotic syndrome)
**Break**	30		
**Small-Group Discussion**	180	Differential diagnosis discussion about a case of pedal edema	Discussion about three diseases (Cirrhosis, CHF, and Nephrotic syndrome)
**Debrief**	30	Open discussion	Open Discussion

The workshop’s goal in the intervention group was to help students develop a correct problem representation from the patient’s clinical problem and organize data into three illness scripts; this was based on a clinical scenario adapted from a study by Levin et al. published in MedEdPortal [13]. .Details on the case and facilitator guide are provided in Supplementary Appendix 1. To help students compare and contrast the findings, the ‘think aloud’ method was also used in the intervention group [14]. An external observer, familiar with the clinical reasoning, observed both intervention and control group workshops, and confirmed using the facilitator’s guide by the facilitator and tutors.

### Evaluation methods

#### Knowledge test

Before conducting the lecture and illness script sessions, the researchers designed ten multiple choice-questions for pretest and posttest. Each question measured a specific teaching point. In both groups, the pretest was done before, and the posttest was done after the teaching sessions. Each question had 2 points, and the total score for each of the pretest and posttest was 20. Both the pretest and posttest were completed on paper in a proctored and closed-book setting. The scores were measured using an answer key that was developed before the administration of the tests. The scorers of the pretests and posttests were blinded to the intervention.

#### Script concordance test

Using SCT development guidelines developed by Bernard Charlin, internal medicine experts at Shiraz medical school developed ten Script Concordance Tests (SCTs) based on the illness scripts of these three important diseases (nephrotic syndrome, cirrhosis, and congestive heart failure) [15]. Our SCT case-based vignettes and questions were designed to evaluate clinical reasoning ability in the diagnostic domain for early medical learners. Each describes a short scenario followed by questions presented in three parts: (1) an appropriate diagnosis option; (2) a new clinical finding; and (3) a five-point Likert scale from - 2 to + 2 that should be chosen by examinees [16]. A sample of SCT is shown in Table 2. We invited ten internal medicine expert faculties to answer the SCT.

**Table 2:** SCT sample scenario. A 50 years old female presents with both lower extremities edema since the last ten days

	If you think about	And you find	This hypothesis becomes
**q1**	**Nephrotic syndrome**	**Proteinuria > 3 g/** 24 **hours**	**−2 -1 0 + 1 + 2**
**q2**	**Liver Cirhosis**	**jaundice**	**−2 -1 0 + 1 + 2**
**q3**	**Congestive heart failure**	**orthopnea**	**-2 -1 0 + 1 + 2**

− 2:The hypothesis is almost eliminated, 1-:The hypothesis becomes less probable

0:The information does not affect the hypothesis

+ 1:The hypothesis becomes more probable, + 2:The hypothesis is almost approved

The correct answer for an SCT was weighted based on expert response. The credit for the best answer was 100%, and credit for other answers was calculated based on the expert panel’s percentage who chose that answer [17]. Each SCT had 1 point, and the total score of all of the SCT was 10. The students in the intervention and control group participated in this SCT 4 weeks after the intervention.

#### Satisfaction survey

After the SCT administration, students crossed over from the intervention group to the control group and vice versa. All medical students in both groups completed a satisfaction questionnaire/survey about the illness script method after participating in the teaching by illness script workshop. The questionnaire had 14 items/questions and was designed based on our previous questionnaires about educational workshops’ satisfaction and one other study in the illness script teaching method [10, 18, 19]. Students rated each item using a Likert scale (1 = strongly disagree to 5 = strongly agree). Medical education experts determined the validity of the questionnaire using the modified Kappa variation coefficient [20]. The modified Kappa coefficient was 0.75, and the reliability was established after a pilot study (r = 0.87).

### Statistical analysis

Data were analyzed with descriptive and analytic statistics such as paired t-test using SPSS, version 16. The alpha level was at 0.05. The effect size was measure by Cohen’s d method [21].

### Ethical consideration

The Ethics Committee approved of Shiraz University of Medical Sciences approved our study by ethical code number IR.SUMS.REC.1397.470 and did not need to register with the Iranian Registry of Clinical Trials. Informed written consent to participate was obtained from all participants. Participants joined the study voluntarily, and their scores remain confidential.

## Results

Of the hundred participating students, 47 (47%) were male, and 53 (53%) were female. The effect size was 2.04 for pretests and posttests in the intervention group, and 1.99 for the control group. There was no difference in pretest scores between the intervention and control group by student t-test (10.87 + 2.49 vs. 9.84 + 2.85, *p* = 0.083) which was suggestive of similar baseline knowledge in both groups. On the knowledge test, the mean posttest scores (14.38 + 2.59 in control group & 15.74 + 2.47 in intervention group) were higher than the pretest scores (9.84 + 2.85 in control group & 10.87 + 2.49 in intervention group) in both groups, and this difference by paired t-test was statistically significant (*p* = 0.0001 in both groups). The comparison of posttest scores by student t-test in the intervention group was significantly higher than the control group (*p* = 0.009). The effect size for the difference between posttests was 0.54 that is a moderate effect size. (Table 3). The intervention group’s mean score was significantly higher on the SCT than the control group (6.12 ± 1.95 vs. 4.54 ± 1.56, *p* = 0.0001).

**Table 3:** Comparison of pretest and posttest in each group by pair T-test and between groups by students T-test

Groups	Number	Mean ± SD	***p***-value	Cohen’s D effect size
**Intervention Group**				
**Pretest**	50	10.87 ± 2.49	0.0001^*^	**2.04**
**Posttest**	50	15.74 ± 2.47		
**Control Group**				
**Pretest**	50	9.84 ± 2.85	0.0001^*^	**1.99**
**Posttest**	50	14.38 ± 2.59		
**Pre-test**				
**Intervention Group**	50	10.87 ± 2.49	0.083^**^	**0.38**
**Control Group**	50	9.84 ± 2.85		
**Post-test**				
**Intervention Group**	50	15.74 ± 2.47	0.009^**^	**0.54**
**Control Group**	50	14.38 ± 2.59		

^*^ Pair t-test, ** Independent t-test

Our satisfaction survey results indicated that the intervention was generally well-received by students (Table 4). Most students (82%) strongly agreed or agreed that the tutor gave them appropriate feedback. Most students (80%) also believed that the illness script method emphasized learning, and 78% of students reported that they were overall satisfied with the workshop. When students were asked how they would improve the workshop, the main suggestion was that the workshop should stress more on the “thinking aloud” approach.

**Table 4:** Results of the medical students’ satisfaction with the illness script workshop

No	Statement	A	B	C	D
1	I gained a good understanding of concepts in the clinical reasoning field	72 (72%)	20 (20%)	6 (6%)	2 (2%)
2	The workshop encouraged me to improve my clinical reasoning ability in the future	69 (69%)	20 (20%)	8 (8%)	3 (3%)
3	The learning objectives of the workshop were clearly defined.	72 (72%)	26 (26%)	0 (0%)	2 (2%)
4	The amount of material delivered in the workshop was reasonable.	70 (70%)	23 (23%)	6 (6%)	1 (1%)
5	The level of difficulty of the workshop was appropriate.	63 (63%)	24 (24%)	12 (12%)	1 (1%)
6	The program was a good learning experience for me	74 (74%)	16 (16%)	9 (9%)	1 (1%)
7	The illness script model was useful for improving my clinical reasoning skills.	61 (61%)	23 (23%)	15 (15%)	1 (1%)
8	This type of education emphasized learning	80 (80%)	12 (12%)	7 (7%)	1 (1%)
9	The tutor gave us appropriate feedback	82 (82%)	14 (14%)	3 (3%)	1 (1%)
10	The “thinking aloud” method helped improve my clinical reasoning skills.	56 (56%)	22 (22%)	16 (16%)	6 (6%)
11	The workshop was boring and wasted my time	4 (4%)	10 (10%)	86 (86%)	0 (0%)
12	The students’ participation was encouraged	74 (74%)	23 (23%)	3 (3%)	0 (0%)
13	I recommend this type of teaching for other sign and symptoms	76 (76%)	18 (18%)	4 (4%)	2 (2%)
14	Overall, I am satisfied with the course.	78 (78%)	14 (14%)	6 (6%)	2 (2%)

A = Strongly agree/Agree, B=Neutral, C=Disagree/Strongly disagree, D = Missing

## Discussion

This randomized controlled trial aimed to identify the effect of teaching clinical reasoning skills for the diagnosis domain based on the illness script method. Despite the illness script workshop’s briefness in the intervention group and the lecturing in the control group, both the illness script method and lecturing appeared effective. Both groups showed significant improvement in posttest scores in comparison with pretest scores. The findings also showed a high effect size between pretests and posttests in both groups. The results that students improved on the posttest are likely unsurprising as most success in clinical reasoning is attributable to knowledge gained [22].

Our study also showed that illness script teaching intervention helps medical students earn better scores in posttest in contrast to the control group. This better score maybe because teaching by illness scripts method helps students recognize the standard and discriminating features in the intervention group better than the control group with a medium effect size between posttests in the intervention and control groups. A study by Linsen et al. about teaching clinical reasoning to first-year medical students showed that this education would increase students’ participation in the learning process [23].

Like the knowledge test, the SCT result showed a higher intervention group score than the control group. However, medical students’ SCT average score in the intervention groups was around 60% of the total possible score, suggesting less proficiency even in the intervention group. These low scores might be due to their first learning experience with the illness script method and less clinical experience.

Other studies have used SCT for the assessment of clinical reasoning skills. The SCT assesses illness scripts’ formation in medical students by comparing their answers on this test to a panel of experts’ responses. In our previous studies, SCT has been described as a valid and reliable assessing tool in the clinical reasoning field [24–27]. Compared to other studies, we had performed SCT 4 weeks after original workshops, which helped to understand retention of knowledge for some time compared to the immediate posttest, which was better in the intervention group than the control group.

Like our study, several studies have shown the effectiveness of teaching clinical reasoning skills during the formal curriculum. Lee et al.’s study about teaching clinical reasoning to medical students showed that the intervention group’s students scored better than the control group on clinical reasoning tests named clinical reasoning problems [18]. Another study by Delavari et al. about the educational strategies inspired by theory in developing illness scripts revealed that the medical students’ scores on clinical reasoning problem tests improved after the intervention [28]. Another study by Keemink et al. about illness script development in medical students showed that case-based teaching would foster the illness script’s richness [29].

The satisfaction questionnaire results showed that the students were satisfied overall with the intervention. They were also satisfied with the tutors’ appropriate feedback and believed that this course would lead to real learning.

Our study’s most important strength was a randomized controlled nature, which can eliminate several confounding factors. Additionally, assessment of learning was done by multiple methods like knowledge test, SCT, and satisfaction survey showed positive results. Another study’s strength was assessing intermediate-term knowledge retention tested by SCT 4 weeks after the original workshop. There are some limitations to the present study. We might have prepared the students for tests during teaching, especially with the same instructor and tutors for both groups; however, we used the help of an external observer to monitor the educational sections to reduce this limitation. Another limitation is that SCT was not a part of baseline testing, and we only used this test for posttests.

Third, while some changes are statistically significant, we cannot ascertain whether these are educationally significant. Because the results are symptom and sign-specific, we cannot conclude that medical students develop better clinical reasoning skills in general, nor can we be sure of the clinical reasoning skills application during actual clinical practice. Third, this study is a single-center study for a specific group of learners with small sample sizes, so generalizability is limited.

## Conclusion

Teaching with illness scripts method provided an effective way to improve students’ clinical reasoning scores for diagnosis domain posttest and SCT. Whether this approach translates to improved clinical reasoning skills in real clinical settings merits further study. Our findings can serve as a rationale for implementing clinical reasoning education modules for undergraduate medical education curricula to empower medical students in clinical reasoning skills.

## Supplementary Information

**Additional file 1.**

## Data Availability

The datasets used and analyzed during the current study are available from the corresponding author on request.

## Abbreviation

SCT: Script Concordance Test
