# Supplementary material for: Teaching clinical reasoning to undergraduate medical students by illness script method: a randomized controlled trial
Source: BMC Med Educ. 2021 Feb 2;21:87. doi: 10.1186/s12909-021-02522-0 (PMC7856771; doi:10.1186/s12909-021-02522-0)
Supplement: Supplementary file 1 — Additional file 1. [file 12909_2021_2522_MOESM1_ESM.docx]

**Intervention group**

**Facilitator guide:**

1. Individually or in small groups, have students read the case and provide a problem representation. Have groups present their problem representations, and discuss as a class, highlighting key features.

**Case:**

A 55-year-old woman presents with one month of worsening swelling in both legs. She has a history of hyperglycemia, well controlled with diet and exercise, with HbA1c of 5.9%. She has no drug or alcohol use, and no significant family or surgical history. On review of systems, she denies headache, shortness of breath, orthopnea, or paroxysmal nocturnal dyspnea, but does note frothy urine for the past 2 weeks. On exam, blood pressure is 130/90, BMI is 18, with normal skin, cardiovascular, and pulmonary exams. She has symmetric 2+ edema of the bilateral lower extremities, to the umbilicus, without erythema. Her abdomen is non-tender, without masses or organomegaly. Initial labs reveal albumin of 2.5 g/dL.

**Initial Problem Representation:**

*Provide a short summary of the case, using semantic qualifiers (e.g. Young/old, acute/chronic, diffuse/localized, mild/severe, etc.)*

**Middle-aged woman**, who presents with **subacute**, **progressive, dependent edema**, **frothy** **urine**, and **hypoalbuminemia**.

1. Individually or in small groups, have students provide a differential diagnosis for the case. As a class, discuss the possibilities, asking students to justify their choices. Have students describe illness scripts for the differential diagnoses provided below.

| **Illness Scripts:** | *Differential #1*  **Nephrotic Syndrome** | *Differential #2*  **Cirrhosis** | *Differential #3*  **Heart Failure** |
| --- | --- | --- | --- |
| **Epidemiology/**  **Predisposing Factors** | Autoimmune disease, infections (HepB, HepC, HIV), cancer, drugs/toxins, diabetes, amyloid, idiopathic above age 50 | Alcohol, viral hepatitis, hemochromatosis, Wilson’s, a1-antitrypsin, primary biliary cirrhosis, primary sclerosing cholangitis, Budd-Chiari, NAFLD | Coronary artery disease, hypertension, toxins, radiation, vitamin deficiency (thiamine), infection (Chagas, HIV), infiltrate (sarcoid, amyloid, hemochromatosis) |
| **Time Course** | Subacute | Chronic | Acute on chronic |
| **Clinical**  **Presentation** | Dependent and periorbital edema, foamy urine, hypertension, hematuria, thrombosis; late anasarca, pleural effusion, and ascites | Jaundice, spider angiomas, ascites, edema, coagulopathy, asterixis, palmary erythema, caput medusae, fatigue | Dyspnea, orthopnea, paroxysmal nocturnal dyspnea, edema, fatigue, altered mental status, cool extremities, Cheyne-Stokes respiration, elevated JVP, crackles, S3, hepatojugular reflux |
| **Pathophysiology** | 1) Primary sodium retention due to renal disease; OR 2) hypoalbuminemia decreases plasma oncotic pressure, leading to intravascular fluid depletion and secondary activation of renin-angiotensin-aldosterone system; renal protein loss | Portal hypertension (increased intravascular hydrostatic pressure) causes fluid leak across capillaries; hypoalbuminemia decreases plasma oncotic pressure; renal activation of renin-angiotensin-aldosterone system | Increased sodium retention via renin-angiotensin-aldosterone activation, as a result of decreased kidney perfusion, leading to increased intravascular hydrostatic pressure |

1. Compare and contrast the illness scripts, and highlight similarities and differences with the initial problem representation. Highlight key and differentiating features.

- This patient’s age places her at risk for all of the above causes, but she doesn’t have many predisposing risk factors other than hyperglycemia
- Edema is a common symptom of all of the above syndromes
- The subacute time-course, with normal cardiac and pulmonary exams makes heart failure a less likely cause of edema
- Lack of predisposing risk factors or other sequelae of liver failure makes cirrhosis less likely

1. Individually or in small groups, develop a list of labs, tests, and imaging. As a class, have students justify how proposed tests will help rule in/out each diagnosis.

**Labs/Tests/Imaging:**

Urinalysis and microscopic exam – for protein, looking for fat-droplets

BUN and creatinine – looking for renal function

Fasting glucose and HbA1C – to evaluate for evidence of recent uncontrolled diabetes

ANA, C3, C4 – for evidence of vasculitis

Lipid profile – which is abnormal in nephrotic syndrome

Urine protein to Cr ratio (or 24-hour urine for protein) – to make diagnosis of nephrotic syndrome

Ultrasound of abdomen – to evaluate for masses (if no other cause for edema is found), kidney size, presence of venous thrombosis

Chest X-ray – to look for heart size, evidence of CHF, metastases from possible breast cancer

Liver enzymes, PT and INR, hepatitis serology-looking for evidence of liver disease (hepatitis B and C can cause nephrotic syndrome)

**Final Problem Representation/Diagnosis:**

**Nephrotic Syndrome** – key features in this case: subacute onset, foamy urine, absence of predisposing risk factors for cirrhosis or heart failure

**This case was adopted with permission from authors: Levin M, Cennimo D, Chen S, Lamba S. Teaching clinical reasoning to medical students: a case-based illness script worksheet approach. MedEdPORTAL. 2016; 12:10445.**

**Control Group**

**Facilitator guide:**

In a large group, give a lecture about edema and its causes in 30 minutes, Nephrotic syndrome in one hour, liver cirrhosis in one hour, and Congestive heart failure in one hour. After 30 minutes break, lead the students to talk freely about different aspects of these three diseases using textbooks and other databases in three hours. The students will be free and can ask their questions.

Don’t present any case to the students and only discuss the diseases.

Discuss disease-oriented evidence and don’t discuss patient-oriented evidence.

Discuss any lab test or imaging separately for each disease.
